# Revision of Carpal Tunnel Release due to Palmaris Longus Profundus

**DOI:** 10.1155/2015/616051

**Published:** 2015-05-14

**Authors:** Lyrtzis Christos, Natsis Konstantinos, Pantazis Evagelos

**Affiliations:** ^1^Euromedica Kyanous Stavros, Vizyis-Vyzantos 1, 54636 Thessaloniki, Greece; ^2^Department of Anatomy, Medical School, Aristotle University of Thessaloniki, P.O. Box 300, 54124 Thessaloniki, Greece

## Abstract

*Purpose*. The palmaris longus profundus has been documented throughout the literature as a cause of carpal tunnel syndrome. We present a case of palmaris profundus tendon removal during the revision of carpal tunnel release. *Method*. During a carpal tunnel release in a 66-year-old woman, palmaris profundus tendon was found inside the tunnel under the transverse carpal ligament, just above the median nerve, but it was left intact. The patient complained of pain in the hand at night and weakness of her hand one month after surgery. We decided on a revision of the carpal tunnel release. The palmaris profundus tendon was found and was removed. *Results*. The patient had a normal postoperative course. Two months later she returned to her normal activities and was asymptomatic. *Conclusions*. When a palmaris profundus muscle is located in carpal tunnel, we recommend its excision during carpal tunnel release. This excision will eliminate the possibility of recurrent compression over the median nerve.

## 1. Introduction

Carpal tunnel syndrome is the most common compression neuropathy in the upper extremity. Many conditions have been associated with the syndrome such as traumatic disorders, tumors, rheumatoid arthritis, diabetes, hypothyroidism, and fluid retention during pregnancy. Rarely have anatomical variants and muscle and vessel anomalies been described as causes of carpal tunnel syndrome [1].

Most of them are malformation of the flexor digitorum muscle [2], anomalous palmaris longus [3, 4], and aberrant origin and anomalies of the lumbrical muscles [5, 6].

The palmaris longus muscle originates from the medial epicondyle of the humerus as do the flexor digitorum superficialis muscle, the flexor carpi ulnaris muscle, and the flexor carpi radialis muscle. The palmaris longus muscle is located just under the skin, the subcutaneous fat and the fascia of the forearm, just above the flexor digitorum superficialis muscle. Normally, it continues into the flexor retinaculum and the palmar aponeurosis.

The anatomical variations of the palmaris longus muscle have been studied in the past [7]. Its topographic relationship with the median nerve makes its anatomical variations a common cause of median nerve entrapment. There are references in literature on median nerve compression caused by the palmaris longus muscle. This muscle can be fleshy or reversed [8–12].

We present a rare case of carpal tunnel syndrome, due to the abnormal position of the palmaris longus in the carpal tunnel, which causes compression of the median nerve. A second release of carpal tunnel with removal of the palmaris longus was performed, since the palmaris longus was not removed the first time.

## 2. Case Report

A 66-year-old woman complained of pain in the right hand at night, hand weakness, and decreased sensation over the distribution of the median nerve. Physical examination revealed weakness of the thenar muscles. Tinel's sign and Phalen's test were positive. Also decreased sensation throughout the median nerve distribution on the volar aspect of the hand was found. The strength of the abductor pollicis brevis was decreased. The patient underwent a nerve conduction velocity study and electromyogram, which found motor and sensory latency of the right median nerve.

**Figure 1:**
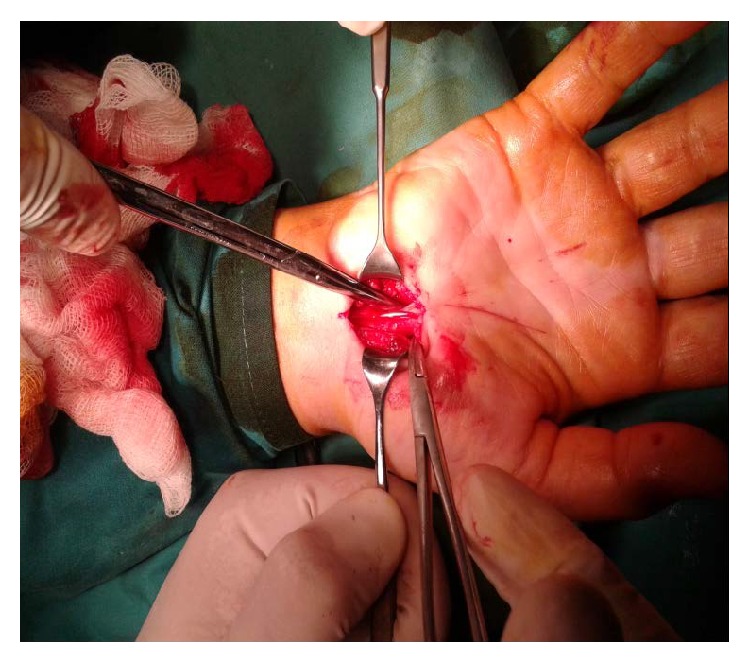
Palmaris profundus in carpal tunnel during the first surgery.

Surgical exploration of the carpal tunnel was performed under local anesthesia without tourniquet control. A local anesthetic numbed the wrist and the hand area. A new 3 cm longitudinal incision on the palmar side in the interthenar region of the wrist was performed from the wrist flexion crease to Kaplan's cardinal line. An incision of the flexor retinaculum was made. The carpal tunnel was entered, and it was found that the palmaris profundus tendon was inside the tunnel under the transverse carpal ligament, just above the median nerve (Figure 1). Severe compression and edema of the median nerve were identified. The palmaris profundus was left intact. The median nerve was verified both proximally and distally. The incision was stitched up with 3 sutures. The hand and the wrist were bandaged.

The symptoms were improved the first night after the surgery, but one month later, the patient visited our clinic. She complained of pain in the hand at night and weakness of her hand. We decided on a revision of the carpal tunnel under local intravenous anaesthesia.

**Figure 2:**
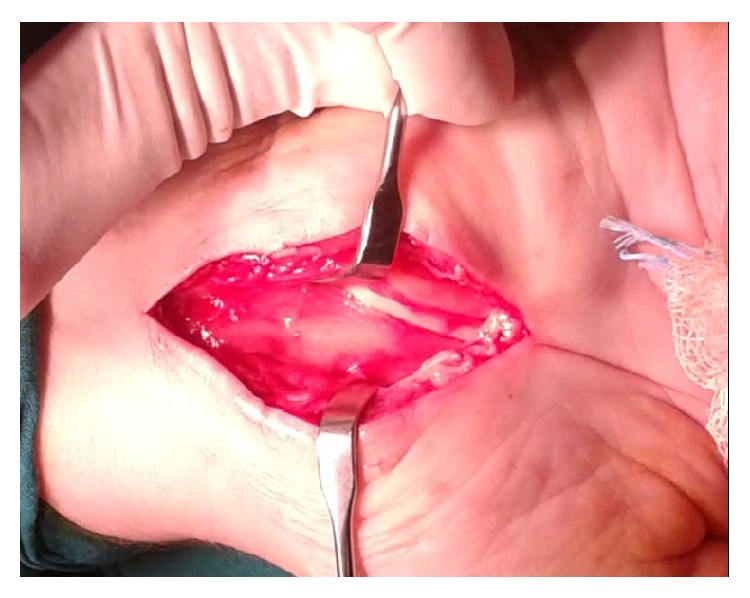
Presence of palmaris profundus over the median nerve during the revision.

**Figure 3:**
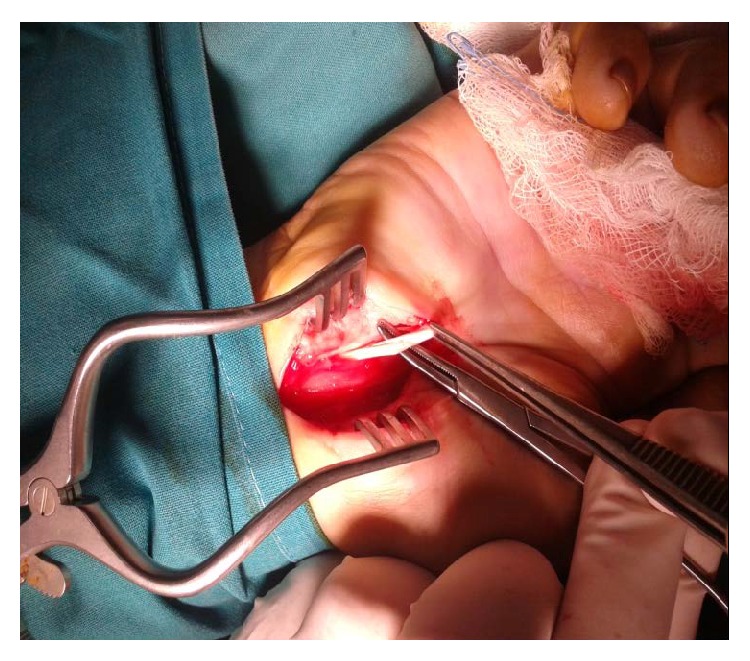
Removal of palmaris profundus after its traction during the revision.

A longer longitudinal incision was performed on the previous one. The palmaris profundus tendon was found and was removed (Figures 2 and 3). The patient had a normal postoperative course. Two months later she returned to her normal activities and was asymptomatic at the several follow-up visits, the last being at 6 months.

## 3. Discussion

Carpal tunnel is an inflexible structure of the wrist. Components which run through this structure are the flexor muscle tendons accompanying their sheath and the median nerve with some of its branches [13]. The median nerve is highly vulnerable to compression in the carpal tunnel. The anatomy of the carpal tunnel is well understood and documented in medical literature. Any structures which pass through the carpal canal can result in compression of the median nerve and cause symptoms of carpal tunnel syndrome.

The abnormal persistent median artery can be a cause of compression of the median nerve. This artery is a branch of the ulnar artery or of the common interosseous artery. It passes through the carpal tunnel of the wrist and may cause carpal tunnel syndrome when it is large or there is an aneurysm, thrombosis, or rupture [1, 14–16].

The origin of the palmaris longus muscle is the medial epicondyle. The tendinous portion begins at the midforearm and inserts distally into the palmar aponeurosis, after passing volar to the flexor retinaculum [17]. Its histological and developmental studies revealed that it has independent origin from palmar aponeurosis [18].

Reimann et al. [7] were the first to study palmaris longus muscles and classify their anatomical variations. Τhe most frequent variation is complete absence of the muscle [17]. The agenesis of the muscles was observed in 12.8% of the cases. The above-mentioned muscles and accessory muscular bundles sometimes replaced the palmaris longus muscle in the occasion of its agenesis. The position of the belly in the muscle can be found in various parts of the forearm. The muscle can be digastric or with a very small belly. Rarely can it be totally muscular or fleshy [12, 19]. Another variation of the palmaris longus muscle is the bifurcation of its tendon or the belly. Double palmaris longus muscle may be associated with the accumulation of connective tissue within the median nerve when it courses through the carpal tunnel [20].

Many cases of reversed palmaris longus muscle have been described in literature. They were found either as an anatomical or as a surgical finding [10, 11, 21, 22]. A reference was made of a three-headed reverse palmaris longus muscle in a female patient who suffered from edema and pain in her wrist that was aggravated during her hand movements. Intraoperatively, a three-headed reversed palmaris longus muscle was found. The patient was free of symptoms after surgery. The correlation between the reversed palmaris longus muscle and the carpal tunnel-like syndrome has been confirmed in literature. Even though the muscle belly passes above the flexor retinaculum, the symptomatology is explained by the compression of the median nerve before its insertion into the carpal tunnel. This condition causes compartment syndrome due to overuse. In the palmar surface of the wrist, the mass that may appear due to the existence of a hypertrophic reversed palmaris longus muscle is a pseudotumor and it can cause problems in differential diagnosis [23].

Another variation of palmaris longus muscle is palmaris profundus. It may exist in addition to the normal palmaris longus muscle [24–26]. If they exist together palmaris longus profundus tends to be deeper than palmaris longus [17]. Its distal tendon passes deep to the flexor retinaculum and inserts on the dorsal aspect of the superficial palmar aponeurosis. The origin of the palmaris profundus muscle is not well known. The presence of the palmaris profundus may be associated with the median nerve compression symptoms [9, 24]. It is difficult to identify the presence of this muscle as the cause of carpal tunnel symptoms. Its presence is at least partially responsible for carpal tunnel symptoms [17]. The failure to adequately recognize this variant may often be the cause of a failed standard carpal tunnel release procedure. The degree of median nerve compression may be associated with the position of the palmaris profundus tendon to its distal insertion. The tendon can be divided and so press the median nerve [27]. Currently, there is no preoperative diagnostic protocol that reliably establishes the presence of palmaris profundus tendon in the setting of carpal tunnel syndrome [17].

In literature there is a case of carpal tunnel released arthroscopically, which was converted to open surgery due to the presence of palmaris profundus tendon [28]. Carpal tunnel syndrome is more common in people with the presence of palmaris longus muscle than in others without this muscle [29]. In literature there is a case of bilateral palmaris profundus muscle coexisting with palmaris longus [9]. Maybe the presence of palmaris profundus muscle increases the risk of carpal tunnel s-m.

## 4. Conclusion

The case described in the present case report is a palmaris longus muscle which caused carpal tunnel syndrome. This case should be taken into consideration in clinical practice by every surgeon and radiologist dealing with the area. Knowledge of the palmaris longus muscle variations and its normal anatomy is useful. The tendon of the palmaris longus muscle is a significant anatomical landmark for surgical approaches in this area and may cause compression of the median nerve. When a palmaris profundus muscle is located in carpal tunnel, we recommend its excision during carpal tunnel release. This excision will eliminate the possibility of recurrent compression over the median nerve, as happened in our case.
