# Corrigendum to “Revision of Carpal Tunnel Release due to Palmaris Longus Profundus”

**DOI:** 10.1155/2017/6038347

**Published:** 2017-11-22

**Authors:** Christos Lyrtzis, Konstantinos Natsis, Evagelos Pantazis

**Affiliations:** ^1^Euromedica Kyanous Stavros, Vizyis-Vyzantos 1, 54636 Thessaloniki, Greece; ^2^Department of Anatomy, Medical School, Aristotle University of Thessaloniki, P.O. Box 300, 54124 Thessaloniki, Greece

In the article titled “Revision of Carpal Tunnel Release due to Palmaris Longus Profundus” [1], the first and last names of all the authors were reversed. The revised authors' names are shown above.
